# Poly(Glycerol Succinate) as Coating Material for 1393 Bioactive Glass Porous Scaffolds for Tissue Engineering Applications

**DOI:** 10.3390/polym14225028

**Published:** 2022-11-19

**Authors:** Eirini A. Nakiou, Maria Lazaridou, Georgia K. Pouroutzidou, Anna Michopoulou, Ioannis Tsamesidis, Liliana Liverani, Marcela Arango-Ospina, Anastasia Beketova, Aldo R. Boccaccini, Eleana Kontonasaki, Dimitrios N. Bikiaris

**Affiliations:** 1 Laboratory of Polymer Chemistry & Technology, Department of Chemistry, Aristotle University of Thessaloniki, 54124 Thessaloniki, Greece; 2 Advanced Materials and Devices Laboratory, Department of Physics, Aristotle University of Thessaloniki, 54124 Thessaloniki, Greece; 3 Department of Prosthodontics, School of Dentistry, Aristotle University of Thessaloniki, 54124 Thessaloniki, Greece; 4 Biohelenika Biotechnology Company, LeoforosGeorgikisSxolis 65, 55535 Thessaloniki, Greece; 5 Institute of Biomaterials, Department of Materials Science and Engineering, University of Erlangen-Nuremberg, 91058 Erlangen, Germany

**Keywords:** glycerol, succinic acid, 1393 bioactive glass scaffolds, dexamethasone, mesoporous nanoparticles, osteogenic differentiation, mechanical properties, bioactivity

## Abstract

Background: Aliphatic polyesters are widely used for biomedical, pharmaceutical and environmental applications due to their high biodegradability and cost-effective production. Recently, star and hyperbranched polyesters based on glycerol and ω-carboxy fatty diacids have gained considerable interest. Succinic acid and bio-based diacids similar to glycerol are regarded as safe materials according to the US Food and Drug Administration (FDA). Bioactive glass scaffolds utilized in bone tissue engineering are relatively brittle materials. However, their mechanical properties can be improved by using polymer coatings that can further control their degradation rate, tailor their biocompatibility and enhance their performance. The purpose of this study is to explore a new biopolyester poly(glycerol succinate) (PGSuc) reinforced with mesoporous bioactive nanoparticles (MSNs) as a novel coating material to produce hybrid scaffolds for bone tissue engineering. Methods: Bioactive glass scaffolds were coated with neat PGSuc, PGSuc loaded with dexamethasone sodium phosphate (DexSP) and PGSuc loaded with DexSP-laden MSNs. The physicochemical, mechanical and biological properties of the scaffolds were also evaluated. Results: Preliminary data are provided showing that polymer coatings with and without MSNs improved the physicochemical properties of the 1393 bioactive glass scaffolds and increased the ALP activity and alizarin red staining, suggesting osteogenic differentiation potential when cultured with adipose-derived mesenchymal stem cells. Conclusions: PGSuc with incorporated MSNs coated onto 1393 bioactive glass scaffolds could be promising candidates in bone tissue engineering applications.

## 1. Introduction

In recent years, polymer science has shown rapid progress in a variety of fields, such as medicine, pharmaceuticals, tissue engineering and industry. The needs of modern life have led scientists to discover new synthetic polymers, without burdening environmental systems [1]. For this reason, they have focused on inventing new alternative methods that avoid the use of toxic catalysts. Synthetic aliphatic polyesters are widely used because of their high biodegradability and cost-effective production [2,3]. The majority of biocompatible polymers which are applicable in medical devices or potentially used in biomedical applications consist of substances that are natural metabolites, biocompatible and nontoxic [4]. Poly(ortho esters) are attractive because of their inherent hydrolytic instability, but simultaneously, these materials with significant functional utility, are difficulty produced, owing to their complex monomer production [5]. Among polyesters, the most investigated is the poly(a-ester) class [6,7]. The attraction of this class of polymers lies in its huge diversity and synthetic flexibility. Poly(a-esters) are usually synthesized by poly-condensation or ring-opening polymerization (ROP) from a variety of monomers [8]. In the last three decades, chemists have been focused on polyglycerol esters due to their ability to modify the pendant hydroxyl group on the polymer chain. Polyesters based on glycerol can possess similar properties to low-mass glycerides, but they would have the benefit of higher mechanical stability and longer in vivo circulation times. These kinds of polyesters are considered to be the most used biodegradable polymers, especially for biomedical and environmental applications, because their short chain in between two ester groups can undergo hydrolysis within a reasonable time frame for biomedical applications, such as drug delivery, sealants or coatings for tissue repair, and as agents possessing antibacterial activity.

Glycerol is a simple trihydric alcohol and is considered to be a derivative of propane; for this reason, it is named 1,2,3-propanetriol. It is colorless, viscous at room temperature, odorless when pure and neutral to indicators [9]. Glycerol serves many roles in the human body, such as the backbone unit of triglycerides and phospholipids, which are the premier form of energy storage and a major ingredient of cell membranes. Its empirical formula, C_3_H_8_O_3_, has a molecular weight of 92.09, and its structural formula shows it to have two primary and one secondary hydroxyl groups, with different reactivity. The primary hydroxyls are usually more reactive than the secondary group, and the first react more readily than the second. However, this generalization does not always apply. Glycerol can form mono-, di-, and triethers. They may be either ethers of glycerol with itself (polyglycerols, PG), inner ether (glycidol), or mixed ethers of glycerol with other alcohols [10]. For these reasons, glycerol-based polymers are very attractive for both fundamental studies and practical applications.

Succinic acid is a four-carbon dicarboxylic acid with the chemical formula HOOC(CH_2_)_2_COOH, and belongs to the category of aliphatic acids. It is a white, odorless solid and is an important bio-based monomer. According to the US Food and Drug Administration (FDA), succinic acid and glycerol can be considered safe materials and have been approved for medical applications. Both these natural compounds are plenteous and their major advantage is their cost-effective production [11,12]. Agach et al. [13] reported petrochemical-free one-step synthesis of poly(glycerol succinate) (PGxS) oligoesters, where x represents the glycerol-to-succinic acid (Gly/Suc) molar ratio, carried out without use of a solvent or catalyst. Carnahan et al. reported the synthesis of PGSuc dendrimers using benzilidene acetal as a protecting group, which can be selectively removed under mild conditions. All dendrimers were found to be amorphous and their glass transition temperatures (Tg) increased from −20 to 14 °C as the molecular weight increased [14]. In a recent work of Valerio et al., the synthesis of PGSuc polyesters using glycerol of different purities and sources was described in order to evaluate the effect of glycerol purity on polyester properties. Polymerization was carried out at 180 °C using different monomer ratios and reaction times [15].

Bioactive glasses, such as 1393 (53 wt% SiO_2_, 6 wt% Na_2_O, 12 wt% K_2_O, 5 wt% MgO, 20 wt% CaO and 4 wt% P_2_O_5_), have been widely used for hard tissue regeneration. These glasses promote strong bonding to tissues through biological apatite formation and enhance osteogenesis [16]. Three-dimensional (3D) porous scaffolds containing a variety of bioactive glasses have been used in hard tissue regeneration for the restoration of bone defects [16]. Their porous structure stimulates cells towards proliferation and differentiation, leading, through a cascade of chemical and molecular events, to in vivo bone formation. 1393 bioactive glass presents optimal viscous flow during sintering, allowing the production of fully dense scaffolds via the foam replica technique, with a smooth surface of struts and pores in the range of 100–500 μm [17,18].

The development of polymeric coatings has been a topic of great interest in recent years, particularly concerning their use for biomedical applications such as tissue engineering [19]. As the porosity of bioceramic scaffolds produced via the foam replica method leads to poor mechanical properties, the fabrication of composite polymer–ceramic scaffolds could be a solution combining the advantages of both high porosity and enhanced mechanical strength; meanwhile, other properties such as antimicrobial or antibacterial ones can also be tailored from polymeric materials [20]. Many approaches have been suggested to enable the controlled release of drugs and/or molecules from composite scaffolds. In this respect, the incorporation of drug-loaded carriers into polymeric matrices and coatings could be a promising strategy for optimizing the drug release profile [21,22,23]. Mesoporous silica-based nanoparticles (MSNs) have been promoted as a promising drug delivery system because of their unique ordered pore structure, with their pores having a 2–50 nm diameter, high surface area and low zeta-potential, which permits high drug loading and release capacity. The incorporation of Ca in the network of MSNs can trigger osteogenic differentiation of stem cells towards extracellular matrix calcification [24,25,26].

Dexamethasone is one of the most widely used corticosteroid drugs for treating a range of dental conditions [27], such as reducing postoperative pain for patients who have had impacted third molars extracted. Most studies have shown that oral dexamethasone reduces pain and swelling after oral surgery. For several decades, dentists have administered corticosteroids before or immediately after surgeries in order to decrease inflammation and other related symptoms. The major action mechanism of dexamethasone is the inhibition of the proteins of the phospholipase A2 group, which has the effect of reducing fluid transfusions, and therefore, edema [28]. However, due to its bulkier and hydrophobic structure, dexamethasone has poor water solubility, and for this reason, its salt, dexamethasone sodium phosphate (DexSP), has been extensively used. In fact, dexamethasone phosphate is a prodrug and is very water soluble, allowing relatively large doses to be administered in a small volume of diluent; moreover, it is inactive until the phosphate groups are broken down by phosphatases in the body to produce dexamethasone.

The aim of the present work is the synthesis of poly(glycerol succinate) (PGSuc) and the evaluation of its efficiency as a coating material for 1393 bioactive glass scaffolds in terms of mechanical properties, degradation, bioactivity, biocompatibility and the osteogenic differentiation of human adipose mesenchymal stem cells (hAMSCs). Neat PGSuc, as well as DexSP-loaded PGSuc and PGSuc with DexSP-loaded MSNs, were evaluated as different coatings for 1393 bioactive glass scaffolds.

## 2. Materials and Methods

### 2.1. Materials

The succinic acid (ACS reagent, ≥99.0%) and glycerol (ACS reagent, ≥99.5%) were purchased from Sigma-Aldrich chemical company (Saint Louis, MO, USA). All the other reagents used were of analytical grade and purchased from Sigma-Aldrich. For the 1393 scaffold fabrication, PVA was purchased from Merck, Darmstadt, Germany and PU foam from Pahlke Schaumstoffe, St. Katharinen, Germany. Dexamethasone sodium phosphate with purity >99.99% was purchased from Pharmathen S.A. (Athens, Greece).

### 2.2. Synthesis of PGSuc

The synthesis was performed in two steps: (a) pre-polycondensation and (b) thermal cross-linking. During polycondensation, proper quantities of glycerol (Gly) and succinic acid (SA) were introduced into a single-neck round-bottom flask (Sigma-Aldrich, Burlington, MA, US) equipped with Dean–Stark apparatus. The mixture was heated at different temperatures—160 °C (PGSuc1), 170 °C (PGSuc2) and 180 °C (PGSuc3) (isothermal reactions)—without the addition of a catalyst, under inert and neat conditions (Table 1). In addition to a COOH/OH molar ratio of 1:1, an additional ratio of 1:2.5 was also tested at 160 °C (PGSuc1b) to see how the reaction proceeded with a different ratio of reactive groups. For the cross-linking step, the pre-polymers, PGSuc1 and PGSuc2, were further kept at 120 °C under vacuum for 24 and 48 h, while those at 180 °C (PGSuc3) were already cross-linked and could not be further handled. After the thermal cross-linking, rubber-like solids with elastic properties were obtained. PGSuc1 was selected for its optimal elastomeric form and its ease of handling.

### 2.3. Characterization of Polymer

#### 2.3.1. Nuclear Magnetic Resonance Spectroscopy

The ^1^H-NMR and ^13^C-NMR spectra of polyesters were obtained by means of an Agilent spectrometer (Santa Clara, CA, USA) operating at a frequency of 400 MHz for protons at room temperature. Deuterated acetone (CD_3_COCD_3_) was used as a solvent to prepare solutions of 5% *w*/*v*. The number of scans was 16, and the sweep width was 6 kHz.

#### 2.3.2. Fourier Transform Infrared Spectroscopy

The FTIR spectra were obtained using a PerkinElmer FTIR spectrometer (Spectrum 1, Waltman, MA, USA) on KBr containing tablets of the samples. One drop of the polymer was subsequently mixed with KBr. By means of a hydraulic press, the mixed powder was mechanically pressed to form a cylindrical tablet. The spectra obtained were in the range of 4000 to 400 cm^−1^, at a resolution of 4 cm^−1^, using 16 co-added scans. All spectra presented are baseline-corrected, normalized and converted to absorbance mode.

#### 2.3.3. X-ray Diffraction

X-ray powder diffraction (XRD) was employed to study the semi-crystalline structure of all samples at room temperature. The XRD spectra were recorded by means of a MiniFlex II XRD system (Rigaku Co., Tokyo, Japan), with CuKa radiation (λ = 0.154 nm), in a 2θ range from 5° to 60°, with a scanning rate of 0.05 deg/min.

#### 2.3.4. Acid Value Determination

After every half hour of reaction, the proper quantity of polymer was removed from the reaction apparatus and dissolved in a THF/MeOH solution. Then, 2–3 drops of phenolphthalein were added into the mixed solution and stirred for a while. A total of 0.1 mol/L potassium hydroxide solution was also used to calibrate the above solution. Thus, the acid value was determined by the following formula:

Acid value = *V* × *C* × 56.1/m

where *V* is the consumption of the titrated KOH solution volume (mL), *C* is the concentration (mol/L), *m* is the quality the polymer (g) and *56.1* is the potassium hydroxide molar mass (g/mol).

#### 2.3.5. Hydrolysis Test

In order to evaluate the spontaneous hydrolytic degradability of polymers, PGSuc samples (after 24 cross-linking) were immersed in 100 mL phosphate buffer (PBS) of pH 7.0 at 37 °C (human body temperature) and were stored in an incubator (MaxQ 4400 incubator, Thermo Fisher Scientific Inc., Waltham, MA, USA) at 100 rpm at 37.0 ± 0.5 °C for 15 days.

### 2.4. Synthesis of 1393 Bioactive Glass Scaffolds

The bioactive glass-based scaffolds were produced via the foam replication method [29]. A slurry was prepared using melt-derived 1393 bioactive glass, poly(vinylalcohol) (PVA, fully hydrolyzed, Mw ~30,000; Merck Millipore, Darmstadt, Germany) and deionized water. PVA was dissolved in deionized water at 80 °C at a concentration of 0.01 mol/L and magnetically stirred for 1 h. Subsequently, the solution was cooled down to room temperature and 1393 BG powder was added slowly under continuous magnetic stirring at a concentration of 40 wt./v% and stirred for 1 h. A 45 ppi polyurethane (PU) foam was used as a sacrificial template (Pahlke Schaumstoffe, Germany). The PU foam was cut and cleaned with acetone in an ultrasonic water bath. For the coating, the foams were immersed in BG slurry for 1 min and manually squeezed to remove the excess slurry, followed by drying at 60 °C for 1 h. Two coatings were applied and left to dry overnight at 60 °C. Moreover, a heat treatment was performed on the samples at a heating rate of 2 °C/min to 400 °C for 1 h in which the PU foam burned out, followed by a BG sintering process at 700 °C for 1 h.

### 2.5. Synthesis of MSNs

The synthesis of silica-based MSNs of MCM-41 type, with a composition of 50 SiO_2_ and 50 CaO% mol, was carried out through a modified sol–gel technique as previously described [24,30]. NaOH was used as an alkaline medium, cetyltrimethylammonium bromide (CTAB) as an agent for the mesoporous structure (soft template), tetraethyl orthosilicate (TEOS) as a Si source and Ca(NO_3_)_2_.4H_2_O as a Ca source. The molar ratio was 1TEOS/0.13CTAB/0.4NaOH/1280H_2_O. The precipitate after washing was dried at 60 °C for 12 h and calcined at 600 °C for 5 h to remove the soft template.

### 2.6. Drug Loading and Release of MSNs

A total of 1 g of MSNs was dispersed in 100 mL of dexamethasone sodium phosphate methanol solution (10 mg/mL) and vigorously stirred for 24 h at 37 °C. After the separation of DexSP-loaded nanoparticles from the suspension with centrifugation at 5000× *g* for 15 min, they were dried in air for 24 h. The mobile phase consisted of ACN/H2O (acidified with phosphoric acid at a final pH of 5) at 40/60 *v*/*v* and the flow rate was 1 mL/min. The drug loading was calculated using HPLC-UV apparatus as described in a previous study [31] and calculated according to the equation:

DL (%) = [weight of drug in nanoparticles]/[weight of nanoparticles] × 100

In vitro drug release studies were performed using dissolution apparatus (Distek, Evolution 2100C, North Brunswick Township, NJ, USA) with an autosampler (DS Evolution 4300 (North Brunswick Township, NJ, USA) using the basket method (USP I method). Drug-loaded MSNs enclosed in a dialysis cellulose membrane bag with a molecular weight cut-off of 12,400 g/mol, were placed onto appropriate sample holders. The dissolution medium was 250 mL of simulated body fluid (SBF) (pH = 7.4, 37.0 ± 0.5 °C) and the stirring rate was kept constant at 50 rpm. The HPLC method was used to assess the dexamethasone content. At predetermined time intervals, 2 mL of the release medium was removed, filtered and assayed, while the experiments were performed in triplicate for each MSN group.

### 2.7. Coating of Scaffolds

#### 2.7.1. Coating with PGSu

After preliminary studies with two different concentrations of polymer solutions in methanol: dichloromethane (1:0.5), 10% and 15%, the latter was selected as the coating material for the 1393 scaffolds because of its better mechanical properties (see results of 3.6 section for 1393PM 15%). The scaffolds were immersed in the solution for 15 min, dried to remove the excess polymer, and then, placed in the oven (Witeg, Wertheim, Germany) for 24 h at 120 °C under high vacuum.

#### 2.7.2. Coating with PGSu + DexSP-laden MSNs

Firstly, 100 mg of the mesoporous nanoparticles (MSNs) was dispersed in 10 mL of DexSP methanol solution (10 mg/mL) and vigorously stirred for 24 h at 37 °C. The DexSP-laden MSNs were separated from the suspension via centrifugation at 5000× *g* for 15 min and were then dried in a high-vacuum oven at room temperature. The resulting drug loading nanoparticles were dissolved in PGSu dichloromethane:methanol solution (1:0.5) in order to prepare a 15% PGSuc + 10% Dexsp MSN mixture solution. The immersion of the scaffolds was performed as described in Section 2.7.1.

### 2.8. Characterization of Scaffolds

#### 2.8.1. Scanning Electron Microscopy (SEM)

The morphology of the scaffolds was assessed via SEM, using an Auriga Base microscope (Carl-Zeiss, Jena, Germany).

#### 2.8.2. FTIR Analysis of Scaffolds

The FTIR analysis of the prepared scaffolds was performed via Fourier Transform Infrared (FTIR) Spectroscopy using a PerkinElmer Spectrometer Spectrum 1000 in transmittance mode in MIR (4.000–400 cm^−1^) with a resolution of 2 cm^–1^ and with 32 scans. Representative scaffolds of all samples were ground into powder to fabricate pellets under pressure (7 tons), with a sample-to-KBr ratio of 1:100.

#### 2.8.3. Mechanical Properties

Compression tests (*n* = 5 per condition) were performed using a single-column universal testing system (Instron 3344, Instron Int. Ltd., Norwood, MA, USA) at room temperature according to ASTM D-695. Scaffolds of approximately 5 mm in diameter and 5 mm in thickness, were placed between the two hardened-steel compression platens, and compression tests were conducted at a crosshead speed of 5.0 mm/min. From the recorded stress–strain lines, the compressive strength and strain were calculated using the formula σ (stress) = F/A, where F is the force and A is the cross-sectional area of the sample.

#### 2.8.4. Apatite-Forming Ability in SBF

Pristine, PGSu-coated, MSN-PGSu-coated, and DexSP-laden MSN-coated 1393 scaffolds were immersed in 75 mL inorganic SBF solution. The solution of the freshly prepared SBF was prepared according to Kokubo’s method and contained ion concentrations that were almost equal to those of human blood plasma [32].

The scaffolds were maintained in an incubator (Incucell 55, BMT Medical Technology, Zábrdovice, Czech Republic) at 37 °C. The solution was replaced at 6 h, 1 day after initial immersion, and then, every 2 days. The scaffolds were collected after 7 and 14 days of immersion, dried at room temperature and investigated using FTIR and SEM (JEOL JSM-7610F Plus, JEOL Ltd., Tokyo, Japan). The pH value was also recorded during the study.

#### 2.8.5. Degradation

The degradation test was carried out using phosphate-buffered saline (PBS pH 7.4) at various time intervals: 2, 4 and 6 h and 1, 2, 3, 7 and 14 days for pH; 7 and 14 days for mass loss. Each sample was weighed before immersion (Wo) and the initial pH (pH1) was measured. For the degradation test, each specimen was placed in 10 mL phosphate-buffered saline (PBS, pH 7.4) and stored in an incubator (MaxQ 4400 incubator) at 100 rpm and 37.0 ± 0.5 °C for the time intervals mentioned above. To measure the dry weight, samples were dried in a vacuum oven for 24 h at 37 ± 2 °C. The difference between the initial mass of samples (Wo) and the mass after the immersion (Wt) provided the initial mass of the degraded sample; thus, the (%) weight loss of the sample was derived using the following equation:

Mass loss = (Wo − Wt)/Wo%

Respectively, the measurement of initial and final pH was carried out using a pH meter (Nahita pH meter, Navarra, Spain).

#### 2.8.6. Cell Culture

The human adipose-derived mesenchymal stem cells (hAMSCs) (passage 2–3) derived via liposuction were provided by Biohellenika SA (Thessaloniki, Greece). hADSCs cell lines were established from adipose tissue specimens according to previously published procedures [33]. The hAMSCs were cultured in Dulbecco’s Modified Eagle Medium (DMEM) (BIOWEST, Nuaillé, France) supplemented with 10% fetal bovine serum (FBS) (BIOWEST, Nuaillé, France), plated onto cell culture plates at 37 °C, 5% CO_2_. The cell culture medium was replaced every other day until 80% confluency, and cell detachment for cell passage was performed using 0.05% trypsin in PBS (BIOWEST, Nuaillé, France).

#### 2.8.7. Biocompatibility

An MTT (3-[4,5-Dimethylthiazole-2-yl]-2,5-diphenyltetrazolium Bromide) metabolic activity assay was performed in 24-well plates, and to do so, early-passage (p3) hAMSCs were seeded at 20,000 cells/well, in triplicate, per experimental condition. The cells were seeded onto the plastic bottom of the wells and left to grow until they reached 80% confluency. At that point, equal amounts of each material in the form of powder were weighed and added to/diluted in the DMEM complete culture medium in each well per condition for 24 h, and incubated at 37 °C, 5% CO_2_. The cytocompatibility/toxicity were assessed the day after via MTT assay. Briefly, the supernatant culture medium was removed and 500 μL of MTT at a concentration of 0.5 mg/mL in DMEM culture medium was added to each well for a −4 h incubation at 37 °C, 5% CO_2_. Upon removal of the MTT, 500 μL/well of DMSO was introduced for 30 min under the same conditions. The reduction in MTT was counted at wavelengths 570/630 nm (PerkinElmer).

#### 2.8.8. Osteogenic Differentiation

The scaffolds were sterilized via autoclaving (exposure to steam at 121 °C, for 15 min, at 115 kPa pressure) before seeding according to previously described conditions [34]. hAMSCs were harvested from the cell culture plates with 0.05% trypsin/0.02% EDTA (BIOWEST, Nuaillé, France). The cells were pelleted via centrifugation at 1000 rpm for 5 min and were resuspended in DMEM supplemented with 10% fetal bovine serum and antibiotics (as described before). The cells were seeded at 3 × 10^3^–2 × 10^4^ onto 96-well plates. The differentiation of hAMSCs was induced using an osteogenic culture medium consisting of complete culture medium; α-minimum essential media (α-MEM) (PAN BIOTECH GmbH, Aidenbach, Germany); 10% FBS (BIOWEST, Nuaillé, France) and antibiotics supplemented with 0.01 μM dexamethasone (Cayman Chemical Company, Ann Arbor, MI, USA); 50 μg/mL L-ascorbic acid 2-phosphate (Cayman Chemical Company, MI, USA); and 10 mM sodium β –glycerophosphate (Cayman Chemical Company, MI, USA). Four experimental groups were created: (1) cell-seeded scaffolds cultured in osteogenic induction medium; (2) a control group of cell-seeded scaffolds cultured in α-MEM supplemented with 10% FBS and 1% penicillin–streptomycin only; (3) cells seeded onto the plastic bottom of the culture plate, cultured in osteogenic induction medium as a positive control for induction; and (4) cells cultured onto plastic in α-MEM supplemented with 10% FBS and 1% penicillin–streptomycin only, as a negative control for induction. The differentiation experiments were performed for 21 days, and the differentiation and control media were changed every second day. The hAMSC-seeded scaffolds were evaluated for the scaffolds’ influence on osteogenic performance through alkaline phosphatase (ALP) activity and alizarin red staining. Moreover, the effect of the treatments on oxidative stress was assessed by determining reactive oxygen species in cell culture supernatants and cell lysates.

- Alizarin red staining (ARS)

ARS is a dye that binds selectively to calcium salts, and it was used to analyze the matrix mineralization. Scaffolds were loaded with 10^4^ hAMSCs (passage 3) in 24-well plates and cultured in osteogenic differentiation medium for 21 days with media replacement every 2 days. Cells cultured with osteogenic or plain culture media were used as controls. To perform the ARS staining, cells in each group were fixed with 10% formaldehyde (Sigma-Aldrich) for 15 min at room temperature (RT) and washed gently twice with PBS. Incubation with 60% isopropanol was performed for 5 min at RT and fixed cells were stained for 1 h with 2% ARS (Sigma-Aldrich). After 3 washes with PBS, the stained cells were lysed in 10 % cetylpyridinium chloride and quantified using a plate reader (PerkinElmer, Waltham, MA, USA) at 405 nm.

- Quantitative measurement of ALP activity and Reactive Oxygen Species (ROS) levels

To determine how the osteogenic capacity of hAMSCs is affected in the presence of each scaffold, an ALP assay was performed. ALP activity was measured in cell culture media and in cell lysates. Cell culture media for ALP analysis were collected at 3, 7 and 21 days of the culture of hAMSC-seeded scaffolds and prepared for the ARS staining experiment described above. For the analysis of cell lysates, 96-well plates were coated with 200 μL of different concentrations of each scaffold, i.e., 17.5, 35 and 70 mg/mL in culture media. After overnight incubation, 100 μL of media was removed and 3 × 10^3^/100 μL passage 3 hAMSCs were seeded on them with and without differentiation media. The cell-seeded scaffolds were maintained in culture for 21 days by changing the media every 2–3 days. After 21 days, the supernatant was removed and stored for further analysis, the cultured scaffolds were washed with PBS twice and the cellular membranes were lysed by adding 100 μL lysis buffer (Tris-HCl 25 mM, TritonX-100 0.5%) at 4 °C for 2 h. After complete lysis, 80 μL lysate or supernatant was aliquoted into another 96-well plate.

Once the samples (cell lysates or supernatant) were placed, an alkaline buffer solution of 1.5 M 2-amino-2-methyl-1-propanol (pH 10.3) was added at 20 μL/well. Afterwards, a substrate solution, prepared by dissolving 100 mg of 4-nitrophenyl phosphate disodium salt hexahydrate in 25 mL of ddH_2_O, was added at 100 μL/ well and incubated for 1 h at 37 °C. The amount of released p-nitrophenylphosphate was estimated by measuring the absorbance at 405 nm using the spectrophotometric method.

The same supernatant and cell lysate samples prepared for the ALP activity were used for ROS determination using SAFAS Xenius (Monaco). The production of reactive oxygen species was evaluated using the cell-permeable ROS-sensitive probe 2′,7′-dichlorodihydrofluorescein diacetate (H2DCFDA), and the fluorescence is expressed in arbitrary units. Each measurement for both the ROS and ALP assays was performed in triplicate.

## 3. Results

### 3.1. Synthesis and Characterization of PGSuc

**Figure 1:**
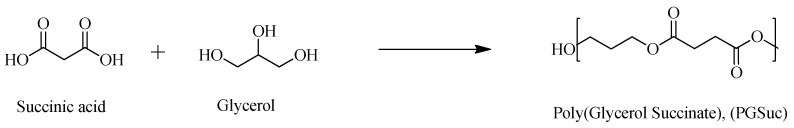
Reaction of succinic acid with glycerol.

PGSuc is a transparent, almost colorless, elastomeric polyester. Its chemical structure is presented in Figure 1. Generally, the pre-polycondensation reaction results in a viscous liquid, except for the case of PGSu1b and PGSu3, where rigid, totally cross-linked polymers were synthesized from the first polycondensation step; thus, they were excluded from further studies. So, it seems that the COOH/OH molar ratio and temperature play a crucial role in cross-linking reactions, and thus, low temperatures such as 160 °C and a COOH/OH molar ratio of 1:1 may be the most appropriate in order to produce viscous and soluble prepolymers.

**Figure 2:**
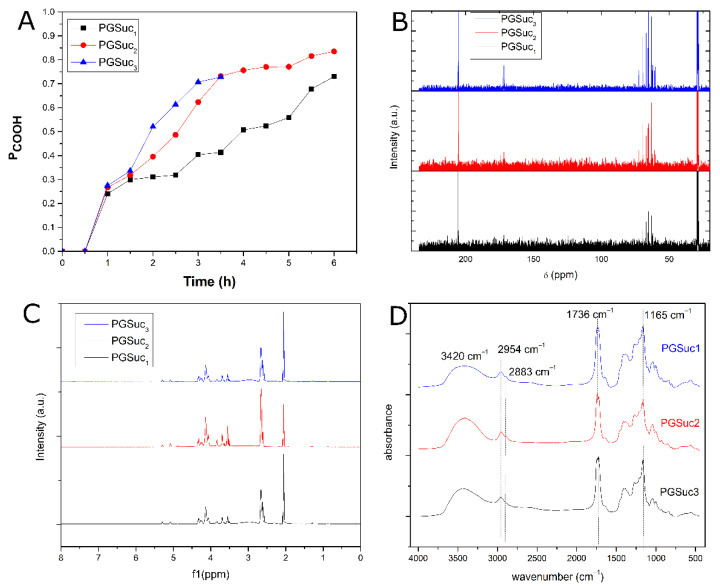
(**A**) Acid value determination, (**B**) ^1^H-NMR spectra, (**C**) ^13^C-NMR spectra and (**D**) FTIR spectra of the polymers.

The progress of the polycondensation reaction was followed by acid value measurements. This is a rapid method in which the evolution of polymerization during the reaction is monitored and is directly related to the consumption of the free carboxylic acid groups of the starting monomers. In this case, the starting monomer is succinic acid. As shown in Figure 2A, in PGSuc3, the esterification was completely carried out in a period of 3 h, in contrast to PGSuc1 and PGSuc2, where the esterification process was executed more gradually than the first. The polymer PGSuc3 was completely cross-linked after a 3 h reaction time, indicating that this temperature is too high. In this case, to avoid cross-linking, the polycondensation reaction should be stopped at lower reaction times. This is also proven by the reaction extent of PGSuc1 and PGSuc2, in which the acid values in PGSuc1 are much lower compared with PGSuc2. So, it is clear that the polycondensation reaction is temperature- and time-dependent; thus, in order to easily handle the prepared prepolymers, it is better to work at temperatures such as 160 °C. Generally, the resulting pre-polymers, PGSuc1 and PGSuc2, were soluble in polar solvents such as methanol. They were insoluble in nonpolar solvents such as toluene and hexane. The resulting prepolymers were typically characterized without further purification. For the cross-linking step, the prepolymers PGSuc1 and PGSuc2 were further heated at 120 °C under vacuum for 24 and 48 h. After this thermal treatment, insoluble rubber solids with elastic properties were obtained for both polyesters and cross-linking times.

#### 3.1.1. ^1^H- and ^13^C-NMR Analysis

NMR analysis combined with FTIR spectroscopy was used to confirm the successful synthesis of polymers at the three different temperatures. The ^1^H-NMR and ^13^C-NMR spectra were obtained at room temperature. ^1^H-NMR (PGSuc) (acetone-d6, 500 MHz) is presented in Figure 2B. For PGSuc_3_, the sample prepared at 3 h was used. Figure 2B depicts the multiplets at 2.66 ppm and represents the –CH_2_ units attached to the carbonyl group (m, CH_2_COOH). Multiplets at 4.03–4.09 ppm represent the glyceryl units (ROCH_2_CHOHCH_2_OR), and methylene units attached to electronegative atoms were observed as multiplets at 4.18–4.37 ppm. The peak at 4.88 ppm corresponds to the –OH protons of PGSuc. ^13^C-NMR poly(glycerol succinate) (acetone-d6, 500 MHz) is presented in Figure 2C. The peak at 173.81 ppm corresponds to the carbonyl ester carbons (C=O) followed by the peaks at 70 ppm showing the carbons connected to oxygen atoms (C–O), indicating the formation of ester groups. The peak at 28.9 is attributed to the carbons connected to carbon atoms (C–C).

#### 3.1.2. FTIR Spectroscopy

The resulting polymer features intense C=O stretching at 1736 cm^–1^ and C–O stretching at 1165 cm^−1^, confirming the successful formation of ester bonds, and thus, the PGSuc prepolymers. The recorded peaks at 2954 and 2883 cm^−1^ are attributed to the stretching of CH_2_ groups on the polymer backbone (asymmetric and symmetric, respectively). The spectrum also shows a broad band at 3420 cm^−1^, indicating that the hydroxyl groups are hydrogen bonded (Figure 2D), probably with the carbonyl groups.

#### 3.1.3. Hydrolysis Test

**Figure 3:**
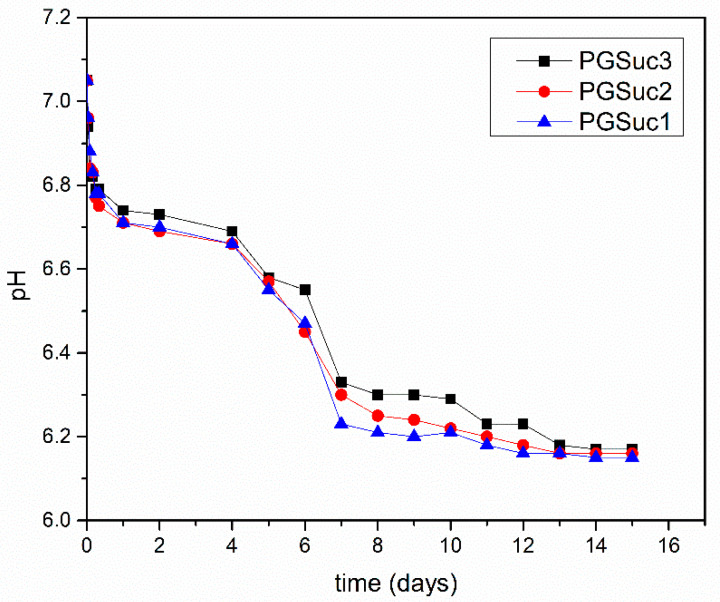
Hydrolytic degradability of PGSuc samples.

PGSuc1 and PGSuc2 elastomers were successfully obtained after cross-linking (24 h under high vacuum at 120 °C) and their hydrolytic degradability was studied in comparison with PGSuc_3_. All cross-linked polymers were immersed in PBS solution (starting pH of 7.05) at 37 °C under intense stirring. As shown in Figure 3, a gradual decrease in pH is observed for all three materials, which is due to the cleavage of the ester bond and the formation of COOH. There is a sharp decrease in pH after day 4 and subsequent stability up to the end of the experiment. Besides the temperature of the synthesis reaction procedure, the hydrolytic behavior of the three kinds of polymers is almost the same. PGSuc1 was selected for coating due to its optimal elastomeric form and its ease of handling.

### 3.2. SEM Analysis of the Scaffolds

**Figure 4:**
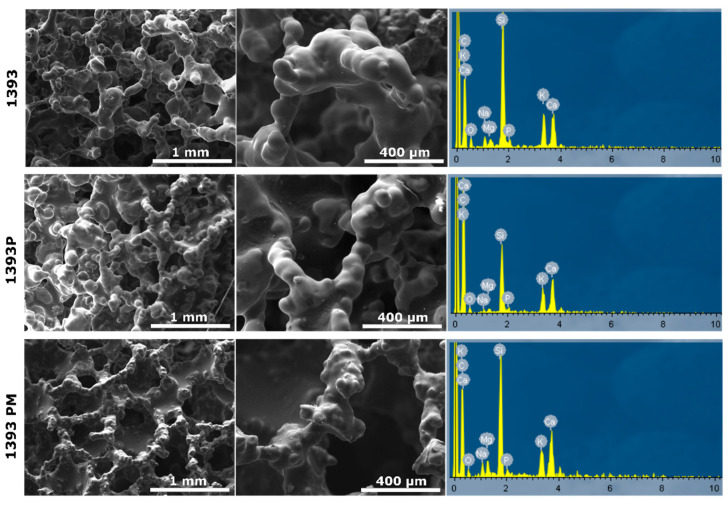
SEM micrographs and EDS spectra of 1393 bioactive glass (1393), PGSuc-coated 1393 bioactive glass (1393P) and MSNs with DexSP-loaded and PGSuc-coated 1393 bioactive glass scaffolds (1393PM corresponding to 1393PM 15%).

Representative micrographs and EDS spectra of coated and uncoated scaffolds are presented in Figure 4.

**Figure 5:**
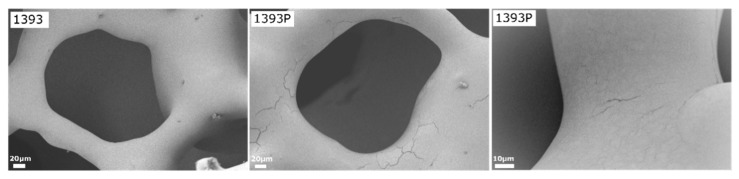
SEM micrographs of uncoated and PGSuc-coated bioactive glass 1393 scaffolds at high magnification (×500). The right micrograph of 1393P is at higher magnification (×2000) and shows surface resembling fabric or film, smoothly covering the scaffold’s struts, with a few areas of minor cracking.

As shown in the micrographs, the uncoated scaffolds present a smooth surface and porous 3D structure, typical of foam replica-derived scaffolds with pores of around 300μm. After PGSu coating, no remarkable differences are observed, although at the higher magnification presented in Figure 5, slight cracking on the scaffold’s struts reveals the presence of the polymer coating. On the contrary, after coating with PGSu with dispersed MSNs, slight sharpening of the scaffold’s surface is seen (Figure 4).

### 3.3. FTIR Analysis of MSNs and Scaffolds

**Figure 6:**
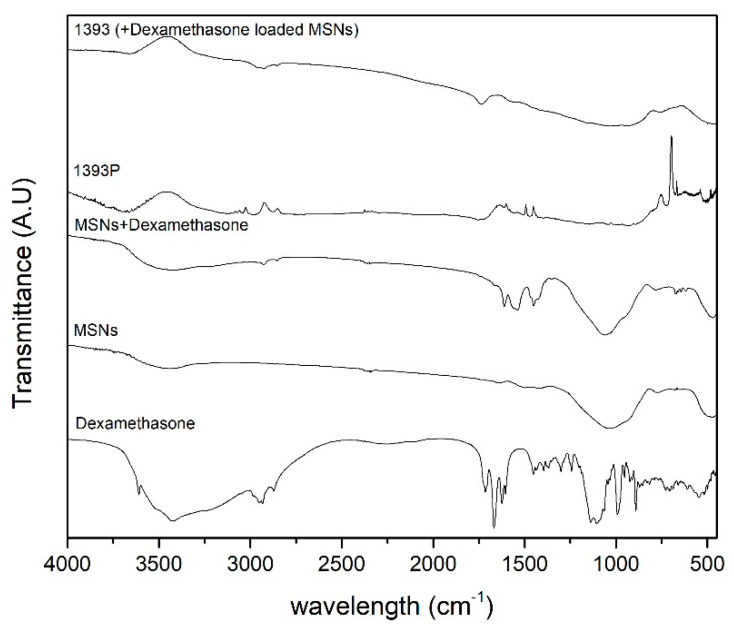
FTIR spectra of dexamethasone, MSNs, loaded MSNs, coated MSNs, and coated and drug-loaded samples.

The spectrum of MSNs represents the characteristic peaks of silicate glasses concerning the contribution of the vibration of the bonds of O–Si in the range of 820 cm^−1^–1330 cm^−1^ (Figure 6). The peak at 508 cm^−1^ is attributed to the vibration of the Si–O–Si bending mode, and the peak at 773 cm^−1^ to the symmetric stretching vibration of Si–O. The vibration of the OH groups appears at 1646 cm^−1^, while the vibration of the appears C–O group at 1424 cm^−1^. Concerning the drug, dexamethasone showed low intensity and a narrow peak at 3613 cm^−1^ (due to free –OH groups), and a broad peak with two maxima at 3520 and 3428 cm^−1^ due to O–H stretching vibration. In addition, the peaks at 1715 and 1668 cm^−1^ are attributed to the vibration of the conjugated (a) and unconjugated (b) carbonyl groups, respectively, while the peaks at 1624 and 1604 cm^−1^ come from C=C stretching vibrations, and the peaks at 1135–1104 cm^−1^ from C–O stretching. The stretching frequency bands of the phosphate anion (P–O) are recorded at 1062 and 1012 cm^−1^, while the band at 992 cm^−1^ corresponds to P–O–C group stretching and deformation vibrations [35].

### 3.4. X-ray Analysis of MSNs and Scaffolds

**Figure 7:**
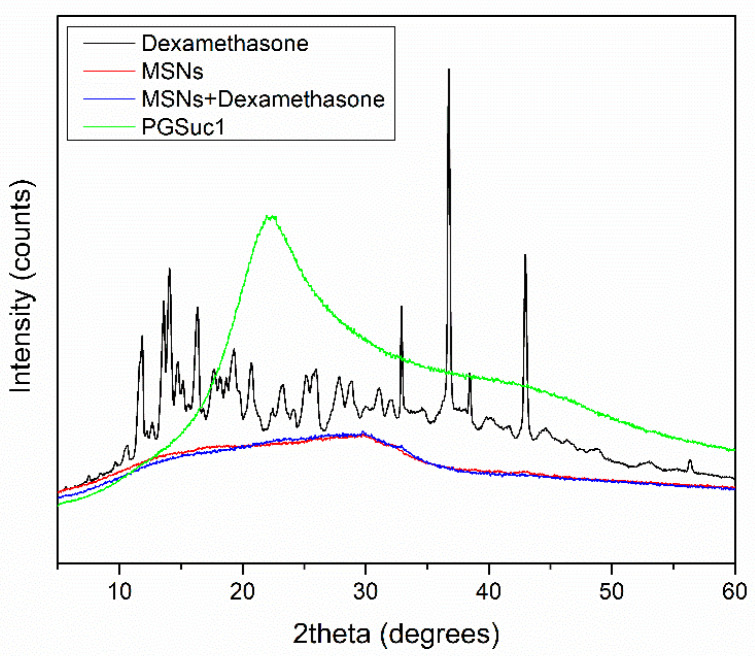
XRD diffractograms of PGSuc1, dexamethasone, MSNs and loaded MSNs.

XRD studies were performed to examine the crystallinity of DexSP before and after its adsorption onto MSNs. Dexamethasone sodium phosphate is highly crystalline with characteristic peaks at 11.82, 14.1, 16.32, 32.82, 36.75 and 42.89 2θ degrees. In the XRD pattern of loaded MSNs, low-intensity peaks were identified as contributing to the amorphization of the drag after absorption (Figure 7). This phenomenon can be attributed to the good dispersion of the drug inside the pores of the MSNs or interactions between the silanol groups of MSNs and the drug [36]. Additionally, with the aid of XRD, the amorphous state of the polymer used for coating was confirmed.

### 3.5. Degradation Study

**Figure 8:**
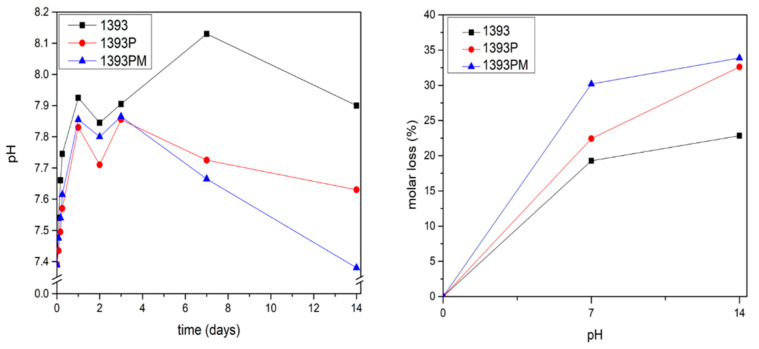
Molar loss of 1393 scaffolds, 1393 coated with PGSuc (1393P), and 1393 coated with PGSuc and MSNs (1393PM).

Figure 8 indicates the changes in pH of the uncoated and coated scaffolds soaked in PBS solution and the respective mass loss. A rapid increase in pH is observed up to the first 3 days for all 1393 bioactive glass scaffolds. The PGSuc-coated (1393P) and PGSuc + MSN (1393PM)-coated scaffolds present lower pH values, compared to those that were uncoated, at all time points, and reach 7.7 and 7.4 after 14 days, respectively. This reduction is due to the hydrolysis of PGSuc and the formation of –COOH, as shown Figure 3. For this reason, the respective mass loss is also higher for the coated scaffolds (~34%).

### 3.6. Mechanical Properties of Scaffolds

**Figure 9:**
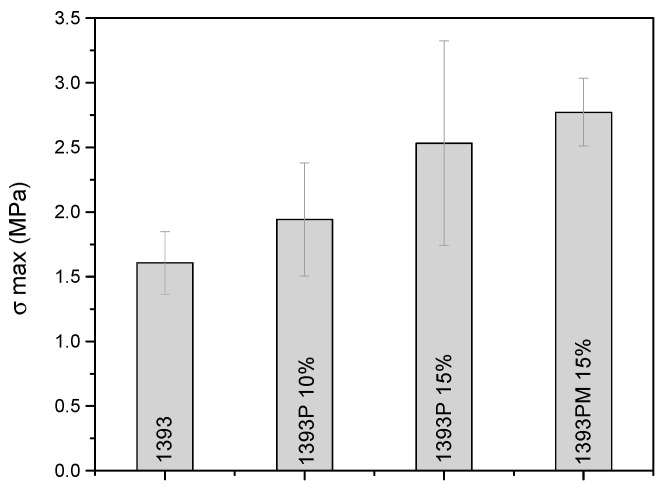
Mean values and standard deviation of compressive strength of 1393 uncoated and coated scaffolds.

Compression tests were performed on the uncoated and coated scaffolds. Figure 9 indicates the values associated with the compressive strength of the samples.

From the compressive strength test, it is evidenced that the increase in the concentration of the polymer in the solution of the coating leads to better mechanical properties (2.54 ± 0.79 for 1393P 15% vs. 1.95 ± 0.43 for 1393P 10%), while the addition of MSNs slightly increases the compression strength (2.77 ± 0.5 for 1393PM 15%).

### 3.7. Apatite-Forming Ability in SBF

#### 3.7.1. Fourier Transform Infrared Spectroscopy (FTIR)

**Figure 10:**
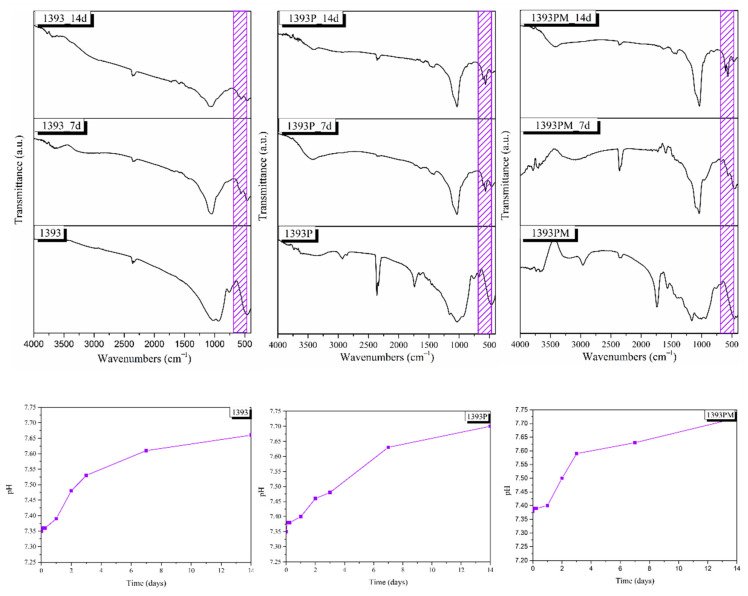
FTIR spectra of all scaffolds after 0, 7 and 14 days of immersion in SBF, and respective pH change. The shaded purple area reveals the presence of the double peak corresponding to the P–O bending vibration.

Figure 10 shows the FTIR spectra of all samples (uncoated 1393 scaffolds, 1393 scaffolds coated with PGSu and 1393 scaffolds coated with MSN-loaded PGSu) obtained after 0, 7 and 14 days of immersion in SBF. The early stages of hydroxyapatite formation involve the formation of a silica-rich layer on the surface of the glasses following cation exchange, which contains Si–OH groups that work as nucleation sites for amorphous calcium phosphate [37,38]. The abrupt increase in pH value in the first days indicates ion release in the solution, while a gradual increase after 7 days, not exceeding pH 7, suggests that calcium ions are incorporated in the precipitated apatite layer [39]. The formation of calcium phosphate (Ca–P) crystalline phases is verified by the characteristic double peak of P–O bending in the IR spectra at around 560 and 600 cm^−1^, and the P–O stretching vibration at around 1035 cm^−1^ [40,41,42]. The FTIR spectra of all samples show P–O bending vibration bands after 7 days of immersion in SBF. The characteristic bands of the carbonate group, CO_3_^−2^, are presented at around 1465, 1422 and 875 cm^−1^, indicating the formation of carbonated apatite (HCA) on the surface of the samples, mimicking bone-like apatite [43,44]. After soaking for 14 days, the spectra of all scaffolds reveal the further growth of carbonated apatite on the surface of the samples, as verified by the sharpening of the double peak at around 560 and 600 cm^−1^, attributed to the P–O bending vibration and the of HCA.

#### 3.7.2. Scanning Electron Microscopy (SEM)

**Figure 11:**
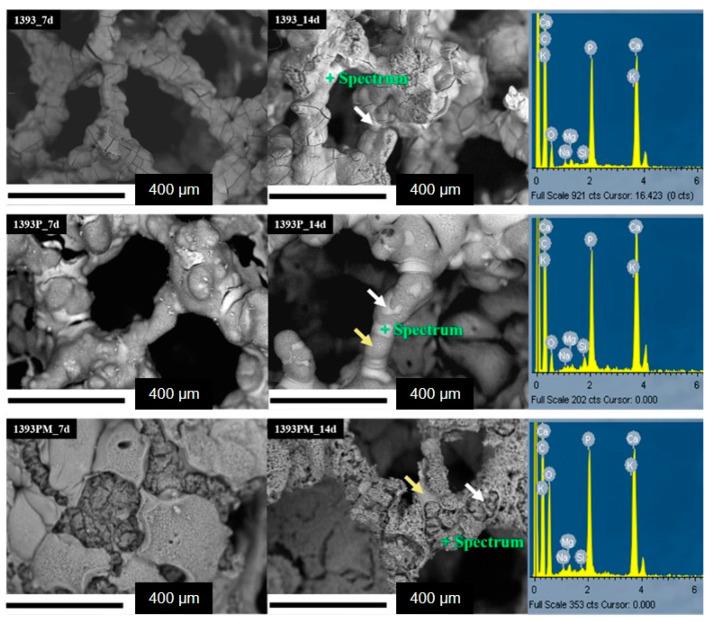
SEM micrographs of backscattered electrons of all scaffolds immersed in SBF for 7 and 14 days and representative EDS spectra of all samples after 14 days of immersion.

SEM backscattered micrographs show the surface morphology of the bioactive scaffolds after immersion in SBF for 7 and 14 days at 37.5 °C (Figure 11). The SEM micrographs of the SBF-treated samples reveal the formation of a uniform HCA-like layer on the surface of the 1393 scaffolds and its presence is verified by the EDS analysis (representative spectra in Figure 11), which shows an increase in calcium and phosphorous concentration with a Ca/P ratio of around 1.6, and a reduction in silicon from the scaffolds; this suggests complete coverage of the scaffold struts with apatite. The formation of apatite is indicated by white arrows. On the other hand, the backscattered micrographs of the composite scaffolds reveal the presence of dark and brighter regions. The brighter regions (indicated by white arrows) are due to the growth of the HCA-like layer, while the dark regions (indicated by yellow arrows) are due to the polymer coating. Moreover, after 7 and 14 days of immersion, degradation of the polymer is also observed, which is in line with the degradation results. These findings are in full agreement with the FTIR analysis.

### 3.8. Drug Loading and Release Studies

**Figure 12:**
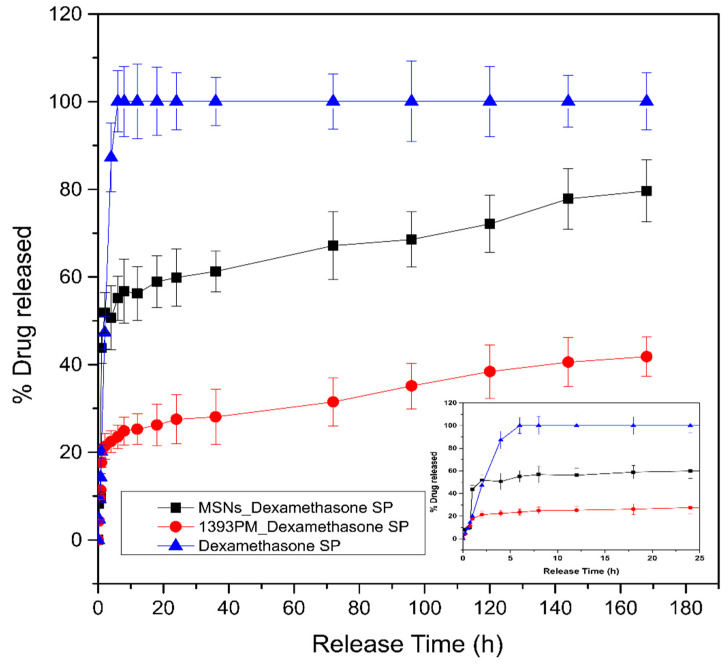
In vitro release study of DexSP from the mesoporous silica nanoparticles. The inset presents the release in the first 20 h.

The drug loading percentage of DexSP was calculated to be 39.02%, while the process was performed in triplicate. The drug release studies of loaded Ca-doped MSNs with dexamethasone sodium phosphate were evaluated and are presented in Figure 12. It is observed that there is a burst release of the neat drug during the first hours, which is attributed to the high solubility of the drug to the aqueous buffer of PBS in which the studies took place. Moreover, the release of the drug encapsulated on the mesoporous nanoparticles seems to present an initial burst in the first hour, followed by a much slower and controlled release. The initial release is a result of remaining drug attached to the surface of the MSNs, while the remaining encapsulated quantity seems to remain inside and is slowly released, presumably due to interactions between the drug and the MSN matrix. In the case of bioactive glass scaffolds coated with PGSuc, the release rate follows a similar profile. A burst release appears in the first release hours, followed by a slower and sustained release until 168 h. This is due to the PGSuc coating, which inhibits the drug release since this release occurs mainly via diffusion and, secondly, via matrix erosion (Figure 3).

### 3.9. Biocompatibility

**Figure 13:**
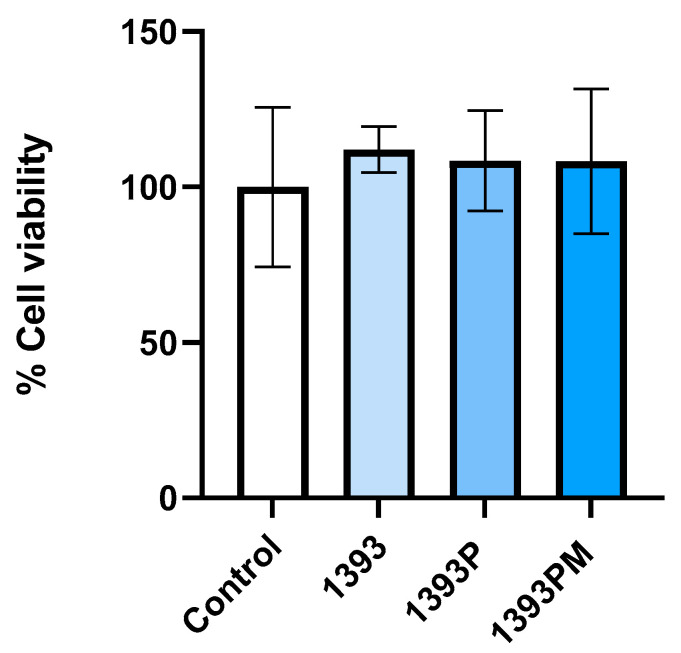
Evaluation of direct contact cytotoxicity of materials based on MTT analysis. The % cell viability corresponds to the ratio of OD (570–630) for each material condition normalized to control (hAMSCs cultured onto plastic) after 24 h of cell culture. Three replicates per condition, ANOVA test, *p* * < 0.05.

The results of biocompatibility testing are presented in Figure 13. Cell viability was evaluated after 24 h of hAMSC culture seeded onto each scaffold via MTT analysis. The results are expressed as relative growth based on calculations, for each condition, of the % ratio of OD (570–630) of the control (hAMSCs cultured onto plastic) at time 0/OD after 24 h of cell culture. The experiment was performed in triplicate; statistical analysis was performed by applying ordinary one-way ANOVA corrected using the Tukey test for multiple comparisons (GraphPad Software, San Diego, CA, USA). As shown in Figure 13, hAMSCs, after 24 h of culture in direct contact with each material, present an increase (although not significant) in the cell growth rate compared to the control, suggesting that all the tested scaffolds are biocompatible.

### 3.10. Osteogenic Differentiation

#### 3.10.1. Alizarin Red Staining (ARS) 

**Figure 14:**
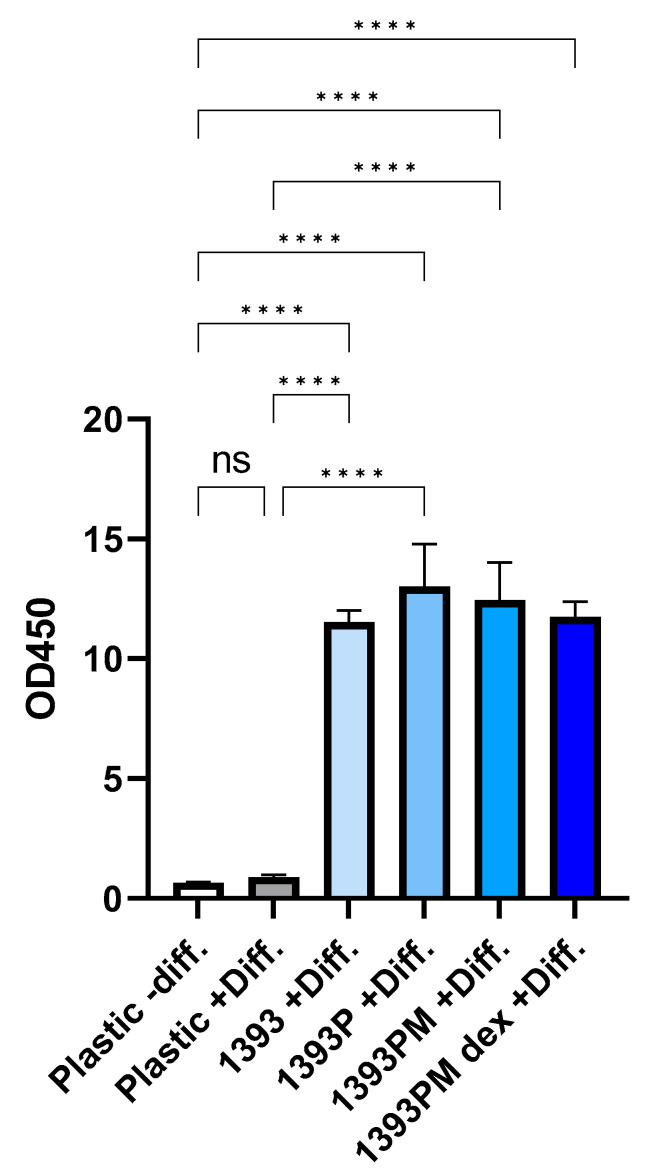
Evaluation of deposition of inorganic calcium salts as osteogenic marker in hAMSCs cultured onto the biomaterials. Quantification of ARS at day 21 was performed by measuring the OD spectrophotometrically at 450 nm. The OD of scaffolds seeded with hAMSCs was calculated after removing the OD of scaffolds subjected to ARS without containing cells. The number of replicates per condition is three; ANOVA test corrected using the Tukey test for multiple comparisons, **** *p* < 0.0001.

ARS was performed to evaluate the presence of mineralized/calcified matrix areas synthetized and deposited onto the scaffolds by hAMSCs. The quantification of ARS was performed by eluting the ARS and measuring the OD. As shown in Figure 14, the deposition of the calcified matrix derived from the hAMSCs grown onto the biomaterials is increased 9- to 11-fold compared to the control + Diff. (*p* * < 0.05, *p* ** < 0.01), implying that all the scaffolds present an osteogenic differentiation potential. It should be noted that the coatings did not suppress the osteogenic differentiation capacity of the 1393 scaffolds, but instead, in 1393P, the deposition of calcium salts is the most evident among all the scaffolds (although not statistically significant). No statistically significant differences are observed among the scaffold groups. Statistical analysis was performed by applying Brown–Forsythe and Welch ANOVA tests for multiple comparisons using the GraphPad Prism software, version 9.3.1 (GraphPad Software, San Diego, CA, USA).

#### 3.10.2. Alkaline Phosphatase Activity (ALP)

**Figure 15:**
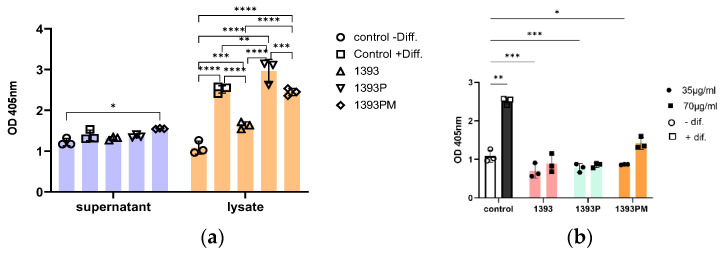
(**a**). ALP activity in hAMSCs cultured in scaffolds on day 21 with osteogenic medium. Quantification of osteogenic differentiation of hAMSCs cultured in scaffolds using alkaline phosphatase assay to analyze cell culture supernatants and cell lysates. Data shown as mean ±SD, and number of replicates per condition are shown on the grap; ANOVA 2-way test corrected using Tukey test for multiple comparisons; * *p* < 0.05, ** *p* < 0.01, *** *p* < 0.001, **** *p* < 0.0001. (**b**) Osteogenic capacity of scaffolds as evaluated via quantification of ALP activity. ALP activity was determined in cell lysates from hAMSCs cultured in scaffolds for 21 days without osteogenic differentiation medium. Different concentrations of materials showing a dose–response effect on the induction of osteogenic differentiation as expressed by ALP activity. Data are presented as means ±SD, and number of replicates per condition are shown on the graph; ANOVA 2-way test corrected using Tukey test for multiple comparisons; * *p* < 0.05, ** *p* < 0.01, *** *p* < 0.001.

Alkaline phosphatase activity is considered a primary indicator of osteoblast secretion, indicating the formation of new calcified tissue. The levels of ALP after 21 days of hAMSCs cultured onto 1393, 1393P 15% and 1393PM 15% scaffolds using osteogenic differentiation medium was investigated by analyzing both cell lysates and conditioned supernatant media. All conditions were compared to ALP activity detected in the hAMSCs cultured on plastic with or without osteogenic differentiation medium. As shown in Figure 15a, the assessment of ALP activity is weak in the cell culture supernatants. The levels of ALP activity seem to be similar among different culture conditions with the exception of 1393PM 15%, where a significant increase is demonstrated compared to the negative differentiation control. The osteogenic potential of the assayed scaffolds is much more apparent when the cell lysates are analyzed (Figure 15a). Our data reveal statistically significant higher osteogenic potential of all the scaffolds, as expressed by ALP activity, compared to the negative differentiation control. Interestingly, 1393P 15% and 1393PM 15% ALP activities are comparable and/or higher than the positive differentiation control, implying that the scaffolds may have per se osteogenic potential. The scaffold 1393P, in particular, presents the best ALP activity performance among all the scaffolds and compared to the +Diff control (*p* = 0.0029). In an attempt to confirm this hypothesis, we repeated this experiment by using plain cell culture medium and exposing the hAMSCs at two different concentrations of the scaffolds (Figure 15b; the highest concentration on this graph is the one used on Figure 15a). As shown in Figure 15b, the ALP activity is induced only when the higher concentration of 1393PM 15% is used compared to the negative differentiation control. However, none of the scaffolds’ osteogenic potential is comparable to the positive differentiation control. There is an indication that higher doses present higher OD values, and this finding merits further investigation.

#### 3.10.3. Reactive Oxygen Species (ROS) Levels 

**Figure 16:**
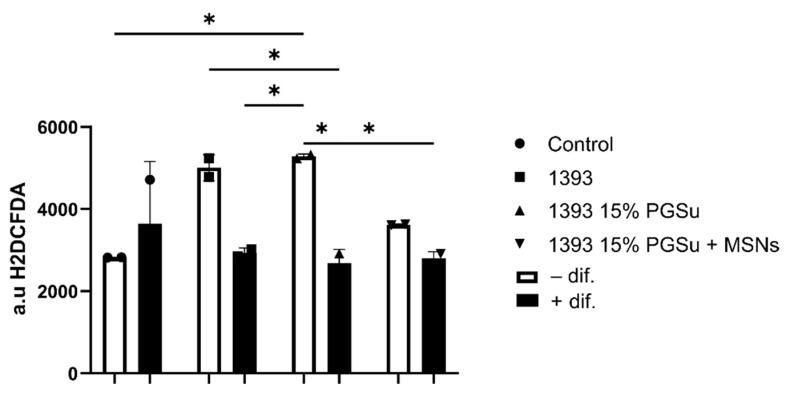
ROS levels as measured from hAMSCs lysates after 21 days of incubation with the scaffolds (70 μg/mL). * = *p* < 0.001.

ROS levels in lysates from hAMSCs after 21 days of incubation with the scaffolds (70 μg/mL) were evaluated (Figure 16). Cells in contact with the scaffolds with differentiation medium produced similar amounts of ROS compared with the ROS generated by the cells without scaffolds. On the other hand, cells in non-differentiated medium presented statistically significant (*p* < 0.001) higher amounts of ROS in contact with the scaffolds compared with the cells alone (CTRL). In Figure 16, the statistical analysis using 2-way ANOVA for multiple comparisons is presented.

## 4. Discussion

Hybrid bone scaffolds are designed to be a temporary supporting structure for bone regeneration in critical-sized bone defects. Scaffolds are supposed to mimic bone composition and architecture with all its complexity from nano- to macro-level, to be biodegradable and to have similar mechanical characteristics to bone. Due to their macroporous structure, scaffolding materials create a favorable environment for the attachment and proliferation of bone progenitor cells within the pores and their osteogenic differentiation into mature bone cells which are able to synthesize a mineralized extracellular matrix. Then, scaffolds are resorbed and gradually replaced by new mineralized bone tissue.

A pivotal role in the performance of a bone scaffold material is assigned to its mechanical properties. Ideally, the mechanical characteristics of bone scaffolds should resemble the ones of native tissue and they should maintain their mechanical integrity throughout the whole duration of regenerative therapy until a new tissue is formed. We investigated the compressive strength of three types of scaffolds and found out that it was comparable to the values of the compressive strength of cancellous bone (1.5–16 MPa) [45,46]. Polymer coating (15%) of the scaffolds led to a significant increase in the compressive strength, while the addition of MSNs did not significantly affect the compressive strength values. Other reported studies found the compressive strength of analogous 1393 scaffolds to be in the range of 2.3 to 18.6 MPa [16,47,48,49,50]. However, the differences in our study could be explained by the different sintering temperature and possibly by the different porosity, different synthesis route and slight differences in composition, i.e., the higher strength for ion-doped 1393 scaffolds.

The PGSuc-coated and PGSuc + MSN-coated scaffolds presented lower pH values compared to the uncoated 1393, due to the presence of PGSuc and silica mesoporous nanoparticles. Since the degradation of polyglycerol esters results in the restoration of carboxylic acid groups, it leads to a reduction in pH values. The reaction of released Ca^2+^ with carboxyl groups further accelerates the dissolution reactions [51]. Silica nanoparticles and MSNs consisting of the infinite Si-O bond can undergo nucleophilic attacks by hydroxide ions (OH^−^) in water to produce silicic acid, Si(OH)_4_, for which silica degradation can be retarded at a reduced pH value of the surrounding environment [52]. The presence of MSNs in the polymeric coating may have accelerated the polymer degradation, or the ion release from MSNs was not enough to provide a buffering effect [53]. It has been reported that pure silica MSNs lose most of their structure after 1 week in PBS [54]. MSN dissolution can promote OH^−^ attacks and further dissolution of the polymer, explaining the higher mass loss of these scaffolds (~34%).

The bioactivity of all scaffolds was tested in SBF and showed the formation of apatite agglomerates after 7 days of soaking in SBF. The early stages of hydroxyapatite formation are known to involve the formation of a silica-rich layer on the surface of glasses following the cation exchange, containing Si–OH groups that work as nucleation sites for amorphous calcium phosphate [37,38]. As reported in the literature, magnesium-containing 1393 bioactive glass shows a significant delay in HA formation compared to other bioactive glass compositions because of presence of magnesium [55]. Interestingly, polymer-coated and MSN-containing scaffolds also presented bioactive behavior. We suppose that the precipitation of HA in this case occurred due to the OH- groups of the polymeric matrix that were released during the first degradation steps of the polymeric coating. The addition of the calcium-doped MNSs appeared to positively affect apatite-formation ability, according to FTIR and SEM/EDS findings. This is in line with the literature, as calcium-containing glasses have the ability to increase the rate of bone-like apatite layer formation [24,56].

The physical absorption of drugs in drug-loaded MSNs follows controlled drug release. According to literature [57], there are two factors that might influence the physical interaction between the drug and the matrix. The first one is the functional groups of the loading system and the second one is the pore wall structure. The presence of silanol groups in the mesoporous channel walls could lead to the formation of weak interactions with drugs, such as electric charge absorption, which keeps the loaded drugs and allows them to be released in a controlled manner [58]. In our case, the interaction between DexSP and the MSNs was gradually destroyed in PBS solution where the dissolution process took place. In general, the slow release rate of the drug from the matrix (PGSuc) and MSNs was due to the fact that PGSuc was dissolved slowly in the aqueous buffer solution. In accordance with the degradation study of 1393PM (Figure 8), the composite lost 30% of its mass during the first 7 days, and until 14 days, the mass loss continued. As a result, we expected a gradual and more sustained release of the drug during the time needed for the complete degradation of the polymer. High concentrations of DEX can decrease cell proliferation and prohibit cell differentiation to the osteoblastic lineage [59,60], not to mention the negative effect on bone tissue and vasculature health with long-term use of glucocorticoids [61,62]. MSNs can favor a sustained DEX release and are able to promote osteogenesis while avoiding side effects. In the present study, we were more interested in studying the release of the drug during the first 7 days, because this is a common time range applied for the systemic administration of most anti-inflammatory drugs. Nevertheless, future work could include studies of its release for more than 7 days, in order to investigate the local concentration of the drug compared to its effective dose for inflammation control. In addition, it would be interesting to investigate the release of the drug molecule in a lower-pH medium, as low pH is a common feature in inflammatory environments. As this procedure is pH-dependent and the release rate is higher at a low pH [63], we expected a greater drug release.

In the current study, hAMSCs were used to investigate early and prolonged in vitro cell–material interactions and to evaluate the materials’ potential ability to promote osteogenic differentiation. hAMSCs have functional and phenotypic characteristics similar to those of bone-marrow mesenchymal stem cells, commonly used in in vitro studies, and have the capacity to differentiate into adipocytes, osteoblasts or chondrocytes depending on the cultivation conditions [64]. Among the advantages of hAMSCs is their minimally invasive isolation from abundant subcutaneous adipose tissue with all the potential benefits for tissue engineering applications.

To evaluate proliferation on the surface scaffolds, an MTT assay was performed at the timepoint of 24 h. The in vitro cell adhesion of specific cell types is closely related to the physiochemical characteristics of the material surface, such as the composition, functional groups, surface charge, topography porosity and surface roughness [65]. The results showed that all of the experimental scaffold types (1393, 1393P, 1393PM) were biocompatible after 1 day of cell cultivation and performed even better than the control, with no statistically significant differences among them. The biocompatibility of 1393 bioactive glass both in vitro and in vivo is well documented in the literature. Due to its hydrophilic silica surface, 1393 bioactive glass may absorb cell adhesion-associated proteins favoring initial cell attachment. This material has been approved by the FDA for its clinical applications. Nevertheless, there are limited data evaluating the biocompatibility of the novel PGSu polymer. Jafari et al. [66] reported that PCL-PGSuc-based scaffolds presented biocompatible behavior and no cytotoxic effects with human corneal epithelial cells (HCE-2), probably because of the enhanced wettability of the produced material. Another author reported that blends of PGSuc and poly(L-lactic acid) (PLLA) and poly(L-lactide-co-caprolactone) (PLCL) supported the cell viability of mouse fibroblast L929 cells [67]. In agreement with their results, we observed favorable behavior of hAMSCs on the surface of the PGSuc-coated scaffold. Importantly, the addition of MSNs did not significantly influence the viability of cells in contact with scaffolds. Our previous study also showed that SiCa50/50 mesoporous nanoparticles supported the proliferation of human periodontal ligament fibroblasts (hPDLFs) [24].

To better characterize our scaffolds, we evaluated their possible osteogenic differentiation properties by performing three-dimensional (3D) cultures of hAMSCs in contact with the experimental scaffolds for 21 days, with or without cell-differentiation medium (depending on the experiment). As reported, when hAMSCs are cultivated in cell-differentiation medium containing vitamins D3 and C, and bone morphogenetic protein-2 (BMP)-2, they transform into osteoblast-like cells within 2 to 4 weeks [64]. Routinely used techniques to assess osteogenic differentiation potential of a scaffold/material include the quantification of ALP activity and ARS staining. ALP activity usually serves as a marker of osteogenic differentiation in the early stages of stem cells’ osteogenic commitment as it progressively decreases when committed osteogenic cells further differentiate towards osteoblast-like cells. Our data provide preliminary evidence that the 1393PM scaffolds at 3 days of culture present the greatest performance upon ALP activity assessment among the scaffolds and compared to the + Diff. control (Figure S1). A gradual decrease in ALP was also observed, in agreement with literature. In the later stages of osteoblast differentiation, cells deposit calcium phosphate into the extracellular matrix, which was visualized using alizarin red staining. The highest increase in ALP activity in the supernatant was detected in the 1393P and 1393PM experimental groups in cell differentiation medium, while the most intensive alizarin red staining was also detected in these groups. We suppose that controllable degradation of the polymer coating during 21 days of observation promoted the slow and sustainable release of Si, Ca, Mg and other ions from the underlying ceramic material, without a dramatic increase in pH; this promoted hAMSC ALP activity and gradual matrix mineralization.

Previous studies have shown that 1393 bioactive glass can induce stem cell differentiation in vitro [68,69], but also present osteoinductive behavior in vivo [70]. It was shown that ionic dissolution products released by bioactive glasses can activate several families of genes associated with osteogenesis or angiogenesis depending on the dosage and material composition. In the current study, we observed increased mineralization of the extracellular matrix induced by 1393 scaffolds, as well as moderate ALP activity; this indicated hAMSC osteogenic differentiation, in agreement with other authors [71]. From all of the groups of materials, 1393 bioactive glass showed the lowest ALP activity. We suppose, that Mg leaching from uncoated ceramics could possibly impair the osteogenic differentiation of ADSC, as reported in the literature [72]. An ICP study would be necessary to confirm this hypothesis.

Another important finding was that 1393PM presented better osteogenic differentiation potential, even when conventional culturing medium was used; this was expressed by significantly higher ALP activity compared to the respective ALP produced by the cells alone. Interestingly, using different concentrations of all the materials promoted a dose–response effect in terms of ALP activity, suggesting biological relevance. As explained in the results section, when scaffolds were cultured without osteogenic medium, they did not induce ALP production at higher levels compared to cells alone cultured in osteogenic medium. Nevertheless, these results are preliminary, and it is important to adjust the cell number-to-scaffold concentration ratio for optimal results in the future. To form a hypothesis about the mechanism by which the scaffolds exert their osteogenic activity, the latter could be explained by the leaching of dissolved Ca and Si ions from MSNs into the surrounding medium and the local increase their concentrations within mesoporous channels; this may stimulate hAMSC differentiation into osteogenic cell lineages [73,74]. However, further investigations of the exact mechanism of the cellular responses are needed. This study has the limitation of not providing the ARS results of cell-seeded scaffolds cultured in conventional medium, in order to form direct conclusions about the osteogenic differentiation potential of each material. However, future studies with a sole focus on the osteogenic capacity of these scaffolds, including molecular and proteomic analysis, would provide further evidence to these preliminary results.

Upon implantation, artificial bone scaffolds can cause oxidant species formation because of the constant oxidative attacks by immune cells or through their degradation products [75]. The production of oxidant species (such as ROS, RNS and LPO) can eventually lead to oxidative stress, when the threshold antioxidant capacity of the surrounding tissues is surpassed [76]. At low levels, intracellular ROS may activate normal cellular processes in hAMSCs and their regenerative potential, while high levels of ROS can cause cellular apoptosis or death [77]. As reported, adipose-derived stem cells are resistant to oxidative stress, and may protect neighboring mature cells from oxidative damage by producing bioactive antioxidant factors (IGF-binding proteins, G-CSF platelet-derived growth factor, superoxide dismutase and HGF) [77]. Our study revealed that ADSTs in contact with scaffold materials generated lower or similar amounts of ROS compared to the control in cell differentiation medium. However, the lowest ROS amount was recorded for the 1393 15% PGSu + MSN scaffold. This may be explained by the ROS consumption by cells during their intensive differentiation. Another finding was statistically significant higher ROS production by cells in contact with experimental scaffolds in plain culture medium compared to cells alone. Such differences can be attributed to the presence of L-ascorbic acid 2-phosphate in cell-differentiation medium, which has strong antioxidant activity.

## 5. Conclusions

In this study, a bio-polyester poly(glycerol succinate) was successfully synthesized and enhanced with dexamethasone sodium phosphate-loaded MSNs as a novel coating material for 1393 bioactive glass scaffolds to develop composite scaffolds for bone tissue engineering applications. The polymeric coating with or without the addition of the MSNs improved the mechanical properties and the apatite formation ability on the surface of the uncoated 1393 bioactive glass scaffolds, increased the degradation rate, and revealed the osteogenic differentiation capacity of adipose-derived mesenchymal stem cells; these are promising results for the application of these scaffolds in different bone regeneration applications.

## Figures and Tables

**Table 1:** Prepared samples based on different temperatures.

Sample	[COOH/OH]	Temperature
PGSuc1	1:1	160 °C
PGSuc1b	1:2.5	160 °C
PGSuc2	1:1	170 °C
PGSuc3	1:1	180 °C

## Data Availability

All data are presented in the article.

## Supplementary Materials

The following supporting information can be downloaded at: https://www.mdpi.com/article/10.3390/polym14225028/s1.
