# Supplementary material for: Poly(Glycerol Succinate) as Coating Material for 1393 Bioactive Glass Porous Scaffolds for Tissue Engineering Applications
Source: Polymers (Basel). 2022 Nov 19;14(22):5028. doi: 10.3390/polym14225028 (PMC9697483; doi:10.3390/polym14225028)

Supplementary data

Figure S1. ALP activity in supernatants of hASCs cultured within the different scaffolds at days 3, 7 and 21 after seeding. Quantification of ALP activity is presented per experimental condition. Data shown as means, ANOVA 2-way test corrected using Tukey for multiple comparisons was used, p*<0.05, p**<0.01, p***<0.001, p****<0.0001

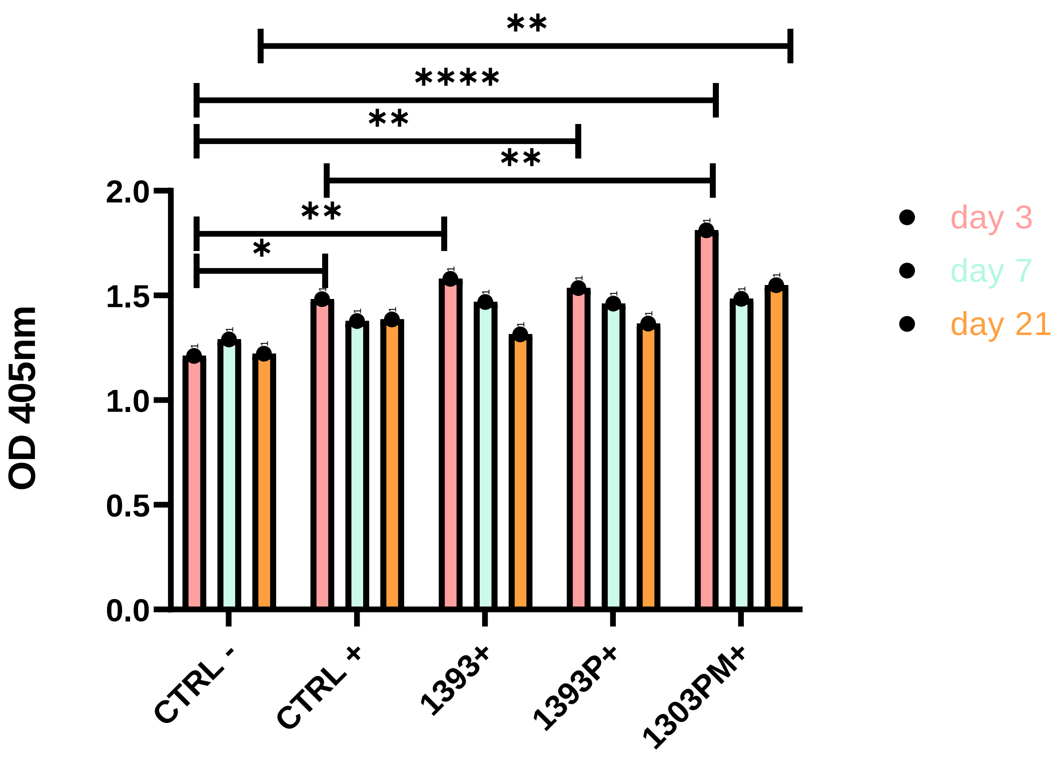

Supplement: Supplementary file 1 [file polymers-14-05028-s001.zip › polymers-1945688-supplementary.pdf]
